# Hydromorphone in Pediatric Pain Management: Pharmacology, Clinical Applications, and Safety Considerations—A Narrative Review

**DOI:** 10.3390/healthcare14142228

**Published:** 2026-07-22

**Authors:** Alessandro Vittori, Cecilia Di Fabio, Ilaria Mascilini, Elisa Francia, Sara Pescatore, Corrado Cecchetti, Giuliano Marchetti, Franco Marinangeli, Teresa Grimaldi Capitello, Marco Cascella

**Affiliations:** 1 Department of Anesthesia, Critical Care and Pain Medicine, ARCO, Ospedale Pediatrico Bambino Gesù IRCCS, Piazza S. Onofrio 4, 00165 Rome, Italy; 2 Department of Life, Health and Environmental Sciences (MeSVA), University of L’Aquila, Piazzale Salvatore Tommasi 1, Blocco 11, Coppito, 67010 L’Aquila, Italy; 3 Surgery Unit, Bios Medical Center, Via Domenico Chelini 39, 00197 Rome, Italy; 4 Department of Neuroscience, Clinical Psychology Unit, Ospedale Pediatrico Bambino Gesù IRCCS, Piazza S. Onofrio 4, 00165 Rome, Italy; 5 Department of Medicine, Surgery and Dentistry, University of Salerno, Via Salvador Allende, 43, 84081 Baronissi, Italy

**Keywords:** hydromorphone, pain, opioid, children, pediatric anesthesia, pediatric pain, chronic pain, addiction, analgosedation, morphine

## Abstract

**Background**: Pain is one of the most common and challenging clinical problems in pediatric care, affecting up to three-quarters of hospitalized children. Hydromorphone has gained increasing clinical interest as an alternative opioid in pediatric settings, although evidence supporting its use remains limited. **Methods**: A narrative literature review was conducted in accordance with SANRA recommendations using PubMed, Embase, and the Cochrane Library to identify studies evaluating hydromorphone use in pediatric and adolescent patients across perioperative, postoperative, intensive care unit (ICU), and emergency settings. **Results**: Available evidence suggests that hydromorphone may provide effective analgesia in pediatric patients across multiple clinical contexts, including postoperative pain control, patient-controlled analgesia, analgosedation in the ICU, and acute pain management in the emergency department. However, the available data are largely derived from small, heterogeneous, and predominantly retrospective studies, often limited to single-center experiences. **Conclusions**: Hydromorphone may represent a useful alternative opioid for pediatric pain management and analgosedation, particularly when morphine is ineffective or poorly tolerated. Nevertheless, the current evidence base is limited, heterogeneous, and largely observational, precluding robust conclusions regarding its comparative effectiveness and safety. Further well-designed pediatric studies are required to better define its role in clinical practice.

## 1. Introduction

Pain is one of the main clinical problems in pediatric care. It is the leading symptom prompting presentation to the emergency department [1] and affects up to 75% of hospitalized children [2,3,4]. Consequently, in pediatric patients, pain management plays a central role in daily clinical practice, with the aim not only of ensuring patient comfort but also of preventing unfavorable medium- and long-term consequences. Nevertheless, despite recent progress, analgesic treatment in children remains a complex topic and is associated with numerous management-related complications. Importantly, the simple extrapolation of data from studies conducted in adult patients is often inappropriate due to physiological differences, organ maturation, and the development of enzymatic systems [5].

When opioid therapy is indicated, morphine represents the gold standard for the treatment of moderate-to-severe pain in pediatric patients, given its well-established efficacy and availability of age-appropriate formulations [6]. Nevertheless, a non-negligible proportion of patients treated with opioids do not achieve adequate analgesia [7]. In cases of insufficient pain control or clinically significant opioid-related adverse effects, therapeutic strategies may need to be reconsidered. In this context, opioid switching may be required to optimize both analgesia and tolerability [8,9,10,11,12]. Consequently, the use of alternative opioids has progressively gained increasing clinical and research interest.

Hydromorphone is a semi-synthetic opioid derived from morphine through hydrogenation and ketone substitution. Introduced into clinical practice in 1926, it shares structural similarities with morphine but exhibits approximately fivefold greater analgesic potency [13]. Chemically, this opioid differs from morphine by the introduction of a keto group at position 6 and the hydrogenation of the double bond between positions 7 and 8 [14]. Interest in hydromorphone has increased in several clinical contexts as a possible alternative to morphine, even though recent meta-analyses do not show statistically significant differences in pain control after surgery between the two drugs [15].

Notably, the literature on the use of hydromorphone in pediatric settings remains limited, particularly in children younger than 6 months [5]. In the absence of pediatric-specific guidelines and robust comparative data, a narrative review was conducted to explore the pharmacological characteristics, clinical indications, and safety considerations of hydromorphone in children. It should be acknowledged that several hydromorphone formulations and indications described in this review are off-label for pediatric patients and should be used in specialized settings under strict monitoring [16].

## 2. Methods

This review was conducted in accordance with the Scale for the Assessment of Narrative Review Articles (SANRA) standard [17]. To ensure comprehensiveness, multiple sources of evidence were integrated. The PubMed, Embase, and Cochrane Library databases were searched using combinations of keywords and Boolean operators related to hydromorphone and pediatric pain management, including: (“hydromorphone”) AND (“children” OR “pediatric” OR “adolescent”) AND (“analgesia” OR “pain management” OR “sedation” OR “intensive care” OR “perioperative” OR “emergency”).

A supplementary search was performed by screening the reference lists of selected articles to identify additional relevant studies. Further literature was identified based on the authors’ clinical expertise and knowledge of the field. Studies published from January 1990 to January 2026 were considered, with priority given to English-language publications.

Studies were considered eligible if they met the following criteria: (i) included pediatric or adolescent populations; (ii) addressed the clinical use of hydromorphone in any setting, including perioperative, postoperative, intensive care, emergency, or chronic pain management; (iii) investigated pharmacokinetics (PK), pharmacodynamics (PD), routes of administration, efficacy, or safety outcomes; and (iv) adopted study designs such as randomized controlled trials (RCTs), observational studies, retrospective analyses, case series, or case reports. Narrative or systematic reviews were considered for contextual purposes but were not used as primary sources of evidence.

Given the limited availability of high-quality pediatric data and the heterogeneity of study designs, results were synthesized qualitatively rather than through formal evidence grading. Data extraction focused on study design, population characteristics, clinical setting, intervention details, and main outcomes, to provide a clinically oriented overview of hydromorphone use in pediatric practice.

## 3. Pharmacokinetics and Pharmacodynamics of Hydromorphone

### 3.1. Elements of Pharmacokinetics and Pharmacodynamics

Hydromorphone exerts its analgesic effect mainly through agonism at μ-opioid receptors, resulting in inhibition of nociceptive transmission at the level of the central nervous system at spinal and supraspinal levels. Opioid receptor interactions determine the analgesic efficacy of hydromorphone [18]. Specifically, hydromorphone has a high affinity for μ receptors, a weak effect on δ receptors, and an absence of clinically relevant activity on κ and ε receptors. This selectivity contributes to defining the PD profile of the drug and to distinguishing it from other opioids used in clinical practice. A PD aspect of particular clinical relevance is represented by the role of the main metabolite of hydromorphone, which is converted through hepatic glucuronidation into hydromorphone-3-glucuronide (H3G) and dihydromorphine-3-glucuronide [19]. Although H3G does not directly contribute to the analgesic effect, several studies have shown that this metabolite has neuroexcitatory properties and is associated with the development of tolerance and hyperalgesia phenomena [20,21]. The clinical relevance of these effects is particularly evident in patients with impaired renal function, in whom accumulation of the H3G metabolite may increase the risk of neurotoxic manifestations, agitation, and tremors. In these cases, the PD of hydromorphone is therefore closely interconnected with its PK and with the body’s ability to eliminate active or potentially toxic metabolites [22]. In pediatric populations, especially in neonates, infants, and critically ill children, age-related renal immaturity or transient renal dysfunction may enhance the clinical relevance of H3G accumulation. Unfortunately, the pediatric studies available to us do not allow for a more in-depth analysis of this topic [23].

### 3.2. Plasma Concentration

The relationship between plasma concentration and analgesic effect represents a further central element of the PD of hydromorphone; specifically, a significant linear relationship (*p* < 0.05) has been demonstrated between plasma levels of the drug and the intensity of analgesia, determining a direct dependence between the clinical effect and systemic exposure [24]. In children, this concentration–effect relationship may be further influenced by developmental PK variability, body composition changes, and differences in opioid sensitivity, supporting the need for careful dose titration and individualized analgesic strategies.

### 3.3. Formulations

The evolution of hydromorphone pharmaceutical formulations has led to the development of extended-release preparations that guarantee more stable systemic exposure and continuous analgesic coverage over 24 h. In particular, extended-release hydromorphone has been extensively studied from a PK perspective. This formulation allows drug release independent of pH and gastrointestinal motility [25] and is relatively little affected by concomitant alcohol intake, reducing the risk of the “dose dumping” phenomenon [26]. Extended-release hydromorphone formulations based on osmotic-controlled release technology allow controlled, once-daily drug delivery, providing stable plasma concentrations and improved tolerability compared with immediate-release preparations. This formulation reaches its maximum plasma concentration between 12 and 16 h after administration, ensuring sustained plasma concentrations for 24 h with dose-proportional PK [25,26,27]. In this formulation, hydromorphone exhibits a longer half-life than the immediate-release form, approximately 13–15 h, and generally reaches steady state after 3–4 days of continuous administration [25,28,29]. Even though overall systemic exposure, evaluated by considering the area under the concentration–time curve (AUC), was similar between the immediate-release formulation and extended-release formulations at the same total daily dose [29], the two preparations differ significantly about the dynamics of plasma concentrations; specifically, the immediate-release formulation is associated with higher Cmax values and more pronounced peak-to-trough fluctuations, whereas the extended-release formulation substantially reduces these oscillations [29]. Moreover, it seems that extended-release hydromorphone shows a significant advantage over the immediate-release formulation by maintaining plasma concentrations ≥50% of Cmax for a much longer period (20.5 h), both after a single dose and at steady state [28,29]. This PK profile makes extended-release hydromorphone suitable in patients with chronic pain who require continuous analgesic coverage over 24 h, reducing plasma fluctuations and consequently the variability of the analgesic effect. However, despite these PK advantages, extended-release hydromorphone formulations have been primarily studied in adult populations, and their use in pediatric pain management remains limited, often off-label, and restricted to carefully selected cases.

### 3.4. Metabolism

The hepatic glucuronidation of hydromorphone to H3G and dihydromorphine-3-glucuronide, without involvement of the cytochrome P450 system, may reduce the risk of clinically relevant drug–drug interactions [19]. From a clinical perspective, assessment of renal function is particularly important before and during prolonged hydromorphone therapy, especially in neonates, infants, and critically ill children, as impaired H3G elimination may increase the risk of neuroexcitatory adverse effects [22].

Although hydromorphone is often considered less susceptible to interactions with enzyme inducers, some studies involving drugs such as rifampin suggest that systemic exposure to opioids may be reduced even in the absence of relevant CYP metabolism [30]. An often-underappreciated aspect in PK/PD studies is the stability of the analyte in biological matrices, as reliable storage and subsequent reanalysis of plasma samples are essential for clinical reassessment and retrospective investigations. In the case of hydromorphone, plasma concentrations have been shown to remain stable in human plasma samples stored at −20 °C for at least three years [31]. The principal differences between adult and pediatric PK/PD characteristics of hydromorphone are summarized in Table 1.

Beyond these pharmacological differences, the clinical implications of hydromorphone therapy also vary according to the stage of physiological development. Practical considerations across the main pediatric age groups are summarized in Table 2.

## 4. Route of Administration

Compared with other opioid analgesics, hydromorphone exhibits notable flexibility in its routes of administration, which allows its use across a wide spectrum of clinical settings and is particularly relevant in the management of cancer pain, as well as acute and chronic pain. Hydromorphone can be administered to patients via intramuscular, intravenous, subcutaneous, rectal, or oral routes [24], allowing analgesic therapy to be tailored to the patient’s clinical condition, the severity of symptoms, and the need for rapid titration or prolonged maintenance of the analgesic effect.

The oral route is considered the preferred route of administration [26,32] and is associated with clear advantages, including safety, convenience, low cost, high patient adherence to therapy, and patient autonomy [32,33,34]. Hydromorphone is available as an oral solution and as immediate-release tablets [35]. In patients who are unable to take medications orally, hydromorphone may be administered parenterally or as suppositories. An injectable formulation of hydromorphone hydrochloride is preferred when it is necessary to administer a high morphine-equivalent dose via the parenteral route [32,36]. The subcutaneous and intravenous routes have been extensively studied both as continuous infusions and through patient-controlled analgesia systems. In the crossover study by Bruera et al. [37], 22 patients with severe cancer pain were treated with subcutaneous hydromorphone infusions for three days either in a patient-controlled mode or as a continuous subcutaneous infusion. More recently, in the study by Lin et al. [38], which included 214 patients with severe cancer pain treated for 24 h, 108 received clinician-controlled intravenous hydromorphone and 106 received patient-controlled intravenous hydromorphone; their findings indicated that the total amount of hydromorphone administered was similar between the groups in both studies while the time to successful titration was significantly shorter in the patients treated with patient-controlled administration compared with those treated with clinician-controlled administration.

The intranasal route has been suggested as an alternative to more frequently employed methods of administration, especially when oral or parenteral routes are challenging. Some benefits of intranasal administration are that it is easy to give, works quickly, and gives patients control. It also increases the drug’s systemic bioavailability by avoiding gastrointestinal degradation and the hepatic first-pass effect [39]. In healthy volunteers, absorption of hydromorphone after intranasal administration was detectable as early as 5 min, and its appearance at the plasma level showed a multiphasic pattern. After 7 intranasal doses of 1 and 2 mg (one every 6 h), mean ± standard deviation peak plasma concentrations of 2.8 ± 0.7 ng/mL and 5.3 ± 2.3 ng/mL, respectively, were observed. The median time to reach peak plasma concentration was 20 min for both single and multiple doses [40]. Following intranasal administration, adverse effects included somnolence, dizziness, and an unpleasant taste. No changes in vital signs were detected; arterial blood pressure and heart rate remained within normal limits, and no respiratory depression occurred. In the pediatric setting, intranasal hydromorphone has been associated with a rapid reduction in pain. Tsze et al. [41] investigated 35 pediatric patients; patients in each dose group experienced an absolute reduction in pain score of more than 3/10 and a percentage reduction of more than 40% within 5–15 min after completion of hydromorphone administration. A duration of analgesia >1 h was observed in 85.7% of patients.

## 5. Clinical Use of Hydromorphone in Pediatric Patients

Although this section focuses on pediatric clinical applications of hydromorphone, readers should note that the available evidence remains heterogeneous and varies substantially across clinical settings. While some perioperative and intensive care data are derived from pediatric observational studies, many pharmacological considerations and several clinical recommendations continue to rely on extrapolation from adult evidence because of the scarcity of well-designed pediatric trials. Consequently, the findings presented in the following sections should be interpreted within the context of the limited pediatric evidence base and should not be considered equivalent to age-specific clinical recommendations. Whenever available, pediatric-specific evidence is explicitly highlighted, whereas extrapolation from adult studies is clearly identified.

**Figure 1:**
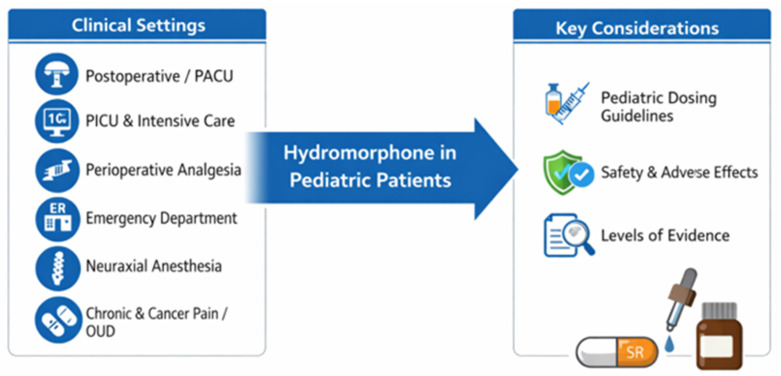
Overview of clinical applications and key considerations for hydromorphone use in pediatric patients. The figure illustrates the main clinical settings in which hydromorphone is employed in pediatric practice, including postoperative care and the post-anesthesia care unit (PACU), the pediatric intensive care unit (PICU), perioperative analgesia, the emergency department, neuraxial anesthesia, and chronic or cancer-related pain. These settings are integrated with key considerations that guide clinical use in children, such as pediatric-specific dosing strategies (e.g., slow release, SR, formulations), monitoring of safety and adverse effects, including the potential risk of opioid use disorder (OUD), and the level of available clinical evidence.

An overview of the main clinical settings and key considerations for hydromorphone use in pediatric patients is shown in Figure 1.

### 5.1. Perioperative Use

The perioperative use of hydromorphone in pediatric patients has been widely documented in clinical practice, particularly for postoperative analgesia, typically as intravenous boluses of 5–10 µg/kg and as patient-controlled analgesia (PCA) [42]. In a prospective observational study [42], 34 pediatric patients undergoing major surgery received standard perioperative care and intravenous hydromorphone boluses (5–10 µg/kg) at surgical closure. Moderate pain was treated with hydromorphone boluses at a dose of 5 µg/kg, while severe pain in the post-anesthesia care unit (PACU) was treated with additional hydromorphone doses (10 µg/kg). Hydromorphone PCA was initiated in the PACU with doses standardized to 4 µg/kg and PCA boluses with lockout intervals of 7–10 min. At the PK level, hydromorphone demonstrates a comparable behavior between pediatric and adult patients when appropriately normalized for body weight. An allometric model using body weight was found to be the most useful in predicting pediatric PK parameters, whereas the other covariates evaluated did not significantly influence PK parameters. Proper titration for individual patients is essential, aiming to maintain hydromorphone concentrations within a safe and effective range. A plasma concentration of 4 ng/mL was identified as the minimum effective concentration associated with analgesia; a plasma concentration of 20 ng/mL produces 50% of maximum pain relief, whereas sedation occurs at concentrations twice this value. Plasma concentrations greater than 51 ng/mL were instead identified in post-mortem analyses of subjects whose death was related to hydromorphone toxicity. In a randomized controlled trial conducted [43] involving pediatric patients undergoing tonsillectomy, hydromorphone was tested as an alternative to fentanyl. Nursing staff in the PACU reported a difference in pain levels between patients treated with hydromorphone and those treated with fentanyl, with the perception that patients receiving hydromorphone required, on average, a lower number of rescue opioid analgesic doses.

Compared with sufentanil, hydromorphone also appears to have a more favorable profile in the control of postoperative pain, as shown in a prospective randomized trial conducted involving 222 pediatric patients undergoing surgery for structural congenital malformations [44]. In this investigation, children were divided into three groups: those who received hydromorphone hydrochloride 0.1 mg/kg (H1), those who received hydromorphone hydrochloride 0.2 mg/kg (H2), and those who received sufentanil 1.5 µg/kg (S). The face, legs, activity, cry, consolability (FLACC) score, a validated behavioral pain assessment tool for pediatric patients [45], was lower in the H1 and H2 groups compared with the S group; the Ramsay sedation score was significantly higher in the H1 and H2 groups compared with the S group, while recovery time was shorter in the H1 group compared with the H2 and S groups. No statistically significant differences were observed with respect to emergence delirium, as assessed using the pediatric anesthesia emergence delirium (PAED), heart rate, respiratory rate, peripheral oxygen saturation, adverse reactions, parental satisfaction with analgesia, or length and cost of hospital stay. The use of PAED alongside behavioral pain scales allowed differentiation between pain-related agitation and true emergence delirium.

### 5.2. Intensive Care Unit and Analgosedation

In critically ill pediatric patients, analgosedation is one of the most complex aspects to manage in the PICU, as it involves patients with a wide spectrum of age, weight, neurological development, and pathophysiological conditions. Children admitted to the PICU are frequently subjected to invasive procedures, mechanical ventilation, continuous monitoring, and repeated handling, all conditions associated with pain, stress, and a significant neuroendocrine response [46]. The primary objective of analgosedation in the pediatric intensive care unit (PICU) is not exclusively patient comfort, but also the reduction in the stress response, the prevention of complications related to PICU stay, and the optimization of clinical outcomes [47,48,49]. Achieving an optimal balance represents a complex and delicate challenge: insufficient sedation may expose the patient to physical and psychological stress, while excessive sedation is associated with prolonged mechanical ventilation, longer PICU stays, and a higher incidence of PICU-related complications [50,51,52,53]. Within this complex clinical context, the “analgesia-first” approach has progressively emerged, the primary aim of which is the prioritization of pain treatment before the introduction of hypnotic or purely sedative agents [54,55]. In mechanically ventilated pediatric patients, opioids can provide effective analgesia, sedation, and modulation of the autonomic response [55,56,57]. Additionally, in the PICU, opioids are especially useful for treating severe, postoperative, and non-neuropathic pain. However, being exposed to them for a long time can be very dangerous. Observational studies have shown that continuous infusions can quickly lead to tolerance and dependence, which can cause withdrawal syndrome when they are stopped [58]. Using opioids with other sedatives, like benzodiazepines, raises the risk of respiratory depression [57,59]. In this complex scenario, hydromorphone has been progressively introduced in the PICU as a drug to be used for continuous infusions, particularly in cases of prolonged sedation, demonstrating effectiveness in providing analgesia and sedation for periods longer than 24 h with an initial dose of 0.024 mg/kg/h and a maximum dose of 0.05 mg/kg/h. Pediatric patients sedated with hydromorphone for a mean duration of 182 h maintained adequate pain control, with 66% of mean daily FLACC scores below 1 [60]. These data suggest that hydromorphone is not only effective in ensuring analgesic efficacy but also allows the achievement of stable and predictable sedation over the long term. On this basis, hydromorphone may represent a particularly relevant option, especially as an alternative to fentanyl, a highly lipophilic opioid that tends to accumulate in tissues, leading to a progressive increase in effective half-life, particularly during prolonged infusions [56,57]. This accumulation may contribute to the development of tolerance and complicate sedation management. The literature suggests that opioid tolerance may be more pronounced with highly lipophilic drugs such as fentanyl compared with less lipophilic molecules such as hydromorphone [57]. Investigations focused on the conversion from continuous fentanyl infusion to hydromorphone in the PICU demonstrated a median reduction of 14% in total opioid dosage, and the median conversion rate was 86%, with a median stabilization dose of 0.08 mg/kg/h [61]. Furthermore, clinical stabilization, characterized by stringent criteria such as the absence of dose modifications, State Behavioral Scale (SBS) scores ranging from 0 to −1 in 80% of evaluations, and diminished reliance on bolus doses, was attained within 24 h in 55.6% of instances, affecting over half of the patients [61]. Sedation achieved through the use of hydromorphone has been evaluated using validated scales; the State Behavioral Scale (SBS) is a central tool for the assessment of sedation in mechanically ventilated pediatric patients [62]. SBS scores range from −3 (unresponsive) to +2 (agitated). The optimal condition for a patient not undergoing neuromuscular blockade is that they are easily arousable or conscious, comfortable, and breathing in synchrony with the ventilator, a state that can be defined as the “Goldilocks Zone” (not too deep and not too light) [51]. This approach is fundamental to reducing the risk of complications such as delirium, neuromuscular weakness, withdrawal syndrome, and other adverse events frequently encountered in the PICU. In critically ill children, renal dysfunction, prolonged infusions, fluid shifts, and concomitant sedatives should be considered when titrating hydromorphone because they may increase metabolite accumulation and the risk of prolonged sedation. Despite the promising results, the literature highlights that the evidence supporting the use of hydromorphone in the PICU is still limited [60], also due to the absence of dedicated pediatric guidelines.

### 5.3. Emergency Setting

Acute pain is certainly one of the most frequent reasons associated with presentation to the pediatric emergency department, particularly due to musculoskeletal trauma and fractures [63]. All of these conditions are often associated with moderate-to-severe pain that requires prompt and effective analgesic treatment. Despite this need, numerous studies have shown that pain management in the pediatric emergency department is still inadequate, with a significant proportion of children receiving no analgesia or receiving insufficient treatment; it has been reported that 35% of children with fractures or severe sprains do not receive analgesia [64,65]. In the emergency setting, opioids continue to represent a fundamental resource for the management of moderate-to-severe pain, albeit within a context of increasing caution related to the opioid crisis. Mild or moderate forms of acute pain can be managed through non-opioid analgesics and non-pharmacological strategies [66]; however, in moderate-to-severe forms, it is often necessary to resort to opioids, always taking into account a multimodal approach. However, the available scientific evidence does not allow the identification of an optimal analgesic strategy for musculoskeletal pain in the pediatric emergency department, also due to the heterogeneity of the drugs and routes of administration that have been studied [65]. Recent research has shown that the addition of oral opioids such as oral morphine to ibuprofen was not more effective and was less safe than the use of oral ibuprofen alone [67]. The use of oxycodone has also not been shown to be safer or more effective than ibuprofen [68]. Tramadol, hydrocodone, and codeine are not recommended for widespread use in children due to safety concerns [69]. Considering these results, oral hydromorphone is being studied as a possible effective alternative to oral morphine and oxycodone [70], thanks to its greater analgesic potency and its duration of action of up to four hours. The intranasal administration of drugs is one of the most interesting routes of administration in the pediatric emergency department, as it allows rapid, effective, and non-invasive analgesia. Hydromorphone, due to its physicochemical characteristics—molecular weight, lipophilicity, and solubility—is favorable for nasal transmucosal administration [71]. The bioavailability of hydromorphone after intranasal administration is approximately 50–60%, with rapid absorption occurring within 5 min of administration [40,72]. Intranasal hydromorphone results in a rapid and clinically significant reduction in pain of greater than 40% within 5–15 min after administration [73]. In the majority of patients (85.7%), analgesia was maintained for more than one hour, and no major adverse events were observed [73], both when administered orally and intranasally, with a safety profile comparable to that of morphine, although based on a still limited body of data. Although hydromorphone can be a valid analgesic option for the treatment of moderate-to-severe acute pain in the pediatric emergency department, especially when incorporated into a multimodal approach and used according to appropriate clinical criteria, further studies are necessary to evaluate its efficacy and tolerability.

### 5.4. Analgesic Adjuvant for Regional and Neuraxial Anesthesia

Hydromorphone has been progressively introduced as an analgesic adjuvant in pediatric regional and neuraxial anesthesia to improve postoperative pain control, reduce the need for systemic analgesics, and optimize safety profiles. In several pediatric surgical settings, the addition of a neuraxial opioid to a local anesthetic has now become a safe and effective clinical practice [74,75]. Opioids acting in the epidural space play a role both at the central level (thanks to their diffusion across the dura mater) and via systemic absorption; all of this results in a reduced response to the surgical stimulus compared with opioid infusion alone or general anesthesia alone [76,77]. The choice of the most appropriate opioid remains a subject of clinical debate, particularly when referring to patient-controlled epidural analgesia (PCEA). Birmingham et al. [75] demonstrated that children older than 5 years are able to successfully manage their own PCEA. However, the opioid agent used is strictly dependent on individual practitioner practice, as the available literature remains limited. Within this context of choice, epidural hydromorphone has been shown to present a more favorable safety profile and lower rates of adverse effects compared, for example, with the use of epidural morphine [78]. In a retrospective review from 2019 [79] conducted on 46 pediatric patients aged between 5 and 17 years, hydromorphone was used in epidural solutions in combination with ropivacaine and compared with epidural solutions containing morphine. The retrospective study showed that PCEAs containing ropivacaine and morphine had a superior ability to control pain compared with PCEAs containing ropivacaine and hydromorphone in the 48 h following surgery, with a greater benefit of morphine compared with hydromorphone observed in the first 24 h after surgery. The use of hydromorphone via the caudal route has been specifically studied in the context of congenital cardiac surgery, where pain control is closely linked to postoperative fast-tracking strategies, which aims at early extubation in the postoperative period, reduction in length of stay, and improvement of postoperative pain control [80]. The use of hydromorphone in caudal injection was associated with “well-controlled” pain on average in 96.3% of patients; the peak pain score remained less than 4/10 for the entire 24-h post-block period in 22% of patients. Approximately 77% of patients who received caudal hydromorphone were extubated in the operating room. Complications related to the caudal puncture were rare and clinically insignificant. The addition of hydromorphone to ropivacaine has been studied in the context of brachial plexus blocks [81]. Hydromorphone can rapidly enter the systemic circulation, reach high blood concentrations, and rapidly diffuse to the brain, heart, liver, kidneys, and other organs, exerting a potent analgesic effect [82]. The adjuvant effect of hydromorphone has been associated with improved postoperative pain control and a reduction in systemic analgesic consumption. Vetter et al. [83] compared hydromorphone, morphine, and clonidine as caudal adjuvants. Morphine demonstrated a longer duration of analgesia, although no statistically significant differences in pain scores emerged during the first 24 h postoperatively. Clonidine was associated with a better tolerability profile, with a lower incidence of nausea, vomiting, and pruritus. Analgesia with hydromorphone, however, was comparable.

An overview of the clinical applications of hydromorphone in pediatric patients, including dosing ranges and levels of evidence, is provided in Table 3. However, these dosages are only indicative since there is no “standard patient” and there are no fixed limits on the maximum dosage of opioids [84].

## 6. Adverse Effects

Hydromorphone is a semisynthetic opioid, structurally and pharmacodynamically similar to morphine but with an analgesic potency 5–7 times greater [5]. Despite its increasing use in pediatric clinical practice, there is a lack of scientific evidence, particularly with regard to safety and the occurrence of adverse events. Despite the rise in prescriptions, the available data remain extremely limited and are completely lacking in children younger than 6 months of age [5]. The recommended dosages for intravenous administration range from 0.01–0.02 mg/kg per dose every 3–4 h or 0.003–0.006 mg/kg/h as a continuous infusion for children older than 6 months of age and weighing more than 6 kg [5].

The adverse effects of hydromorphone are related to activation of μ-opioid receptors. Opioids in general may lead to the occurrence of several side effects, including sedation, urinary retention, pruritus, urticaria, and constipation [85]. A fundamental aspect, confirmed by pediatric trials, is the absence of statistically significant differences between hydromorphone and morphine when used at equianalgesic doses via PCA for the treatment of postoperative pain [86,87]. Sedation is one of the most common side effects of opioid treatment. This is especially important for children because it affects how they are monitored and how safe they are. Available data suggest that hydromorphone may even be more sedating than morphine [23], while acknowledging the overall paucity of pediatric-specific information. In a controlled trial comparing morphine and hydromorphone in postoperative patient-controlled analgesia in pediatric patients, no significant differences were observed between the treatment groups [88].

Respiratory depression is one of the most feared adverse effects associated with opioid use, particularly in children, and is considered rare but extremely serious when it occurs [89]. Hydromorphone itself does not appear to be the sole factor responsible for respiratory depression, which seems to emerge primarily in the presence of an overall sedative burden in patients who had received potentially sedative drugs during recent anesthesia [90]. In these patients, it is mandatory to monitor respiratory and hemodynamic parameters—especially after each administration—and to have available a rescue dose of naloxone appropriate to the patient’s characteristics.

Nausea and vomiting are among the most common adverse effects and have a significant impact on the quality of hospitalization; in children treated via PCA, the incidence is particularly high, with 74% of patients reporting nausea and 43% vomiting (with a peak within the first 24 h). Pruritus occurs in 50% of children within 48 h [88]. From a pathophysiological standpoint, one of the differences between morphine and hydromorphone concerns histamine release; pruritus and hypotension frequently occur after a rapid bolus of morphine in association with histamine release [91]. Despite the absence of a similar histamine-release mechanism with hydromorphone, the clinical frequency of pruritus remains comparable between morphine and hydromorphone. Urinary retention is a less frequent but still clinically relevant postoperative adverse effect, occurring in 8% of pediatric patients treated with morphine or hydromorphone via PCA [88], suggesting the absence of statistically significant differences between the two drugs. A particularly relevant aspect concerns the metabolism of hydromorphone, whose main metabolite, hydromorphone-3-glucuronide, is considered pharmacologically inactive from an analgesic standpoint but not devoid of adverse effects; for this reason, slow titration may be required, as accumulation of H3G can lead to adverse effects such as myoclonus and delirium unrelated to the analgesic effect [92]. Current data are not sufficient to demonstrate a true clinical advantage of hydromorphone over morphine; therefore, hydromorphone should be considered an alternative option to be used in selected settings and under close clinical monitoring.

Concerning opioid use disorder (OUD), opioid dependence represents one of the major public health emergencies in high-income countries [93]. In response to the increase in overdose cases, some settings have introduced safer supply programs, defined as interventions that provide people who use opioids acquired from the illicit market with prescribed pharmaceutical opioids [94]. Within the SAFER program, access to prescribed opioids allowed participants to reduce their use of substances from unregulated markets and to manage withdrawal symptoms, pain, and craving [95]. In specialist clinical settings, hydromorphone has been evaluated as a possible alternative opioid agonist therapy in cases of OUD in which first-line treatments are not feasible [96]. For more severe and treatment-refractory forms of OUD, the use of supervised parenteral hydromorphone (SIOT) has been proposed as a second-line therapeutic option for individuals who do not benefit from standard opioid agonist therapy [97]. Participants in the SAFER program (Victoria Safer Alternatives for Emergency Response) were individuals who utilized opioids sourced from unregulated markets. The program provided prescribed opioids, such as oxycodone and hydromorphone, which are short-acting pharmaceutical opioids. The goal of using these drugs was not to stop using them completely, but to stabilize [95]. A key factor in hydromorphone addiction is getting the right dose; the SAFER program makes sure that people get the right dose and the right drugs. In a patient with OUD stable on methadone therapy, which was discontinued due to QTc prolongation and after failure of sustained-release morphine, sustained-release oral hydromorphone was introduced, the administration of which did not show significant adverse effects [95,96,97,98]. The medication presents an additional advantage in terms of cardiac stability, as it has no influence on the QTc interval. However, in order to prevent misuse or diversion, the use of a 24-h formulation administered via daily witnessed ingestion at a pharmacy, in combination with random urine drug testing, should be considered.

The adverse event profile of hydromorphone in pediatric patients, including the potential risk of opioid use disorder, is summarized in Table 4.

## 7. Perspectives

The growing clinical interest in hydromorphone reflects the need for alternative opioid strategies in pediatric pain management, particularly in patients who do not achieve adequate analgesia or experience unacceptable adverse effects with morphine. From a pharmacological perspective, this opioid offers several potentially advantageous features, including higher analgesic potency, predictable PK, lack of cytochrome P450 metabolism, and multiple routes of administration. These characteristics make hydromorphone an appealing option across heterogeneous pediatric settings, ranging from perioperative care and the emergency department to prolonged analgosedation in the PICU.

On the other hand, the current body of evidence remains fragmented and largely derived from observational studies, retrospective analyses, and extrapolation from adult data. Available research on pediatric populations is generally observational and retrospective, with a small sample size. However, this issue affects all evidence on pediatric pain treatment [99,100], making it difficult, if not impossible, to precisely categorize differences within different age groups. Additionally, significant knowledge gaps persist, particularly concerning optimal dosing strategies across different age groups, long-term safety, neurodevelopmental outcomes, and comparative effectiveness versus other opioids within multimodal analgesic protocols [101,102,103]. In this complex scenario, evidence is especially scarce in neonates and infants younger than 6 months, a population characterized by unique PK/PD vulnerabilities. However, this is a common scenario when discussing pain therapy in pediatrics, where evidence is scarce and limited [99]. Consequently, hydromorphone is frequently used off-label in pediatric patients, reflecting a broader pattern observed across many drug classes in this population [104].

Future research should prioritize well-designed, prospective pediatric trials aimed at defining age-specific dosing, therapeutic plasma concentration ranges, and standardized outcome measures for both analgesia and adverse effects. Comparative studies of hydromorphone and other commonly used opioids, including morphine and fentanyl, are warranted to better delineate its role within analgesia-first and opioid-sparing strategies. Moreover, further investigation into intranasal and neuraxial applications may expand non-invasive and opioid-sparing approaches in acute care settings. A better understanding of hydromorphone pharmacology in children has relevant implications not only for analgesic efficacy but also for safety, opioid stewardship, and the prevention of avoidable adverse outcomes in vulnerable pediatric populations.

Finally, future advances in pediatric pain management may also benefit from model-informed precision dosing and artificial intelligence-based clinical decision support systems. By integrating patient-specific variables such as age, body weight, renal function, concomitant medications, and physiological monitoring, these approaches could assist clinicians in optimizing hydromorphone dosing while minimizing adverse events. Moreover, future pediatric pain management may increasingly integrate digital health technologies, including gamified pain assessment tools, remote monitoring, and model-informed dosing strategies, to improve patient engagement, optimize analgesic therapy, and support individualized clinical decision-making [105]. However, dedicated pediatric validation studies are required before these tools can be implemented in routine clinical practice.

The main research gaps and future directions for hydromorphone use in pediatric patients are summarized in Table 5.

## 8. Conclusions

Hydromorphone could represent a valuable and versatile opioid option for the management of moderate-to-severe pain and analgosedation in pediatric patients. Its use is particularly promising in opioid rotation. However, the limited quantity and heterogeneity of pediatric-specific data preclude definitive conclusions regarding its superiority or preferential use over established opioids. At present, hydromorphone should be considered an alternative opioid to be employed in selected clinical scenarios, particularly when morphine is ineffective or poorly tolerated. Moreover, in neonates, the lack of dedicated PK data currently precludes hydromorphone from being recommended as a first-line opioid, limiting its use to highly selected cases within experienced centers. Therefore, robust pediatric-focused research is required to refine its clinical positioning, optimize dosing regimens, and ensure long-term safety in vulnerable pediatric populations. Until robust pediatric PK/PD and comparative effectiveness studies become available, hydromorphone should primarily be regarded as an alternative opioid rather than a first-line agent in most pediatric clinical settings.

## Figures and Tables

**Table 1:** Main PK/PD differences between adult and pediatric patients receiving hydromorphone.

PK/PD	Adults	Pediatric Patients	Clinical Implication
Absorption	Well characterized for oral, intravenous, subcutaneous, and intranasal formulations.	Greater variability, particularly in younger children because of developmental gastrointestinal physiology.	Careful dose titration is required, especially after oral administration.
Distribution	Relatively stable body composition and volume of distribution.	Higher total body water, lower fat content in infants, and age-dependent body composition changes influence drug distribution.	Weight- and age-adjusted dosing is necessary.
Protein binding	Stable plasma protein concentrations.	Reduced plasma protein concentrations in neonates and infants may increase the free drug fraction.	Potentially enhanced pharmacological effects in younger patients.
Metabolism	Hepatic glucuronidation is well characterized, producing hydromorphone-3-glucuronide (H3G).	Glucuronidation pathways mature progressively during infancy and childhood.	Greater interindividual variability in drug clearance during early life.
Renal elimination	Efficient elimination of H3G in patients with normal renal function.	Renal immaturity in neonates or transient renal dysfunction may reduce H3G clearance.	Increased risk of metabolite accumulation and neuroexcitatory effects.
Plasma concentration–effect relationship	Linear concentration–analgesia relationship demonstrated.	Relationship may be influenced by developmental PK variability and age-dependent opioid sensitivity.	Individualized dosing and close monitoring are recommended.
Analgesic response	Generally predictable in opioid-naïve adults after appropriate titration.	Greater variability in opioid sensitivity and analgesic response across age groups.	Frequent clinical reassessment is essential.
Extended-release formulations	Supported by extensive PK and clinical data.	Limited evidence; use remains largely off-label and restricted to selected patients.	Immediate-release formulations are generally preferred in pediatric practice.
Drug interactions	Limited CYP-mediated interactions; renal function remains clinically relevant.	Similar metabolic pathway, but physiological maturation may modify overall exposure.	Monitor concomitant therapies and organ function carefully.

**Table 2:** Practical clinical implications of hydromorphone PK/PD across pediatric age groups.

Pediatric Population	Main PK/PD Characteristics	Practical Clinical Implications
Neonates (<28 days)	Immature hepatic glucuronidation, reduced renal clearance, high interindividual variability	Use only in highly selected cases; start with conservative dosing and close cardiorespiratory monitoring.
Infants (1–12 months)	Progressive maturation of metabolic pathways and renal function	Frequent dose reassessment; monitor for prolonged opioid effects and metabolite accumulation.
Children (1–12 years)	More predictable PK after weight normalization, although variability persists	Weight-based dosing with individualized titration and regular pain assessment.
Adolescents (>12 years)	PK increasingly resembles adults, although developmental variability remains	Adult dosing principles may be applicable after appropriate weight adjustment and clinical monitoring.
Critically ill children (any age)	Organ dysfunction, prolonged infusions, altered distribution, and reduced metabolite clearance	Individualized dosing, careful monitoring for prolonged sedation, respiratory depression, and H3G accumulation.

**Table 3:** Clinical applications of hydromorphone in pediatric patients.

Setting	Route	Dosing Range Reported in the Literature *	Main Clinical Findings	Evidence
Postoperative/PACU	IV bolus, IV PCA	IV bolus 5–10 µg/kg; PCA bolus 3–4 µg/kg	Effective postoperative analgesia; comparable pain control to morphine; reduced rescue opioid requirement vs. fentanyl or sufentanil in selected studies	Prospective observational studies; small RCTs
Perioperative analgesia	IV bolus	5–10 µg/kg	Adequate intra- and early postoperative analgesia; pharmacokinetics comparable to adults after weight normalization	Observational studies
Pediatric intensive care unit	Continuous IV infusion	0.024–0.05 mg/kg/h	Stable long-term analgosedation; possible alternative to fentanyl with potentially lower tolerance and tissue accumulation	Retrospective cohort studies
Emergency department	Oral, intranasal	Oral: individualized; intranasal 1–2 mg (weight-adjusted)	Rapid pain reduction (>40% within 5–15 min); non-invasive administration; analgesia lasting >1 h	Prospective observational studies
Regional/neuraxial anesthesia	Epidural, caudal	Variable (as adjuvant)	Improved postoperative analgesia; pain control comparable to morphine; acceptable safety profile	Retrospective studies; small trials
Chronic or cancer-related pain	Oral, IV, SC	Individualized equianalgesic dosing	Effective option for opioid switching; flexibility of administration routes	Observational studies; case series
Opioid use disorder *	Oral SR, parenteral (supervised)	Individualized	Used as alternative opioid agonist therapy in selected settings; pediatric-specific data lacking	Adult studies; case reports

Legend: * Reported dosing ranges reflect heterogeneous study designs and should not be interpreted as standardized pediatric dosing recommendations. Opioid requirements vary considerably according to patient age, clinical condition, previous opioid exposure, and individual response; therefore, dosing should always be individualized and guided by clinical monitoring. Abbreviations: IV, intravenous; PCA, patient-controlled analgesia; PACU, post-anesthesia care unit; SC, subcutaneous; SR, sustained release; RCT, randomized controlled trial.

**Table 4:** Adverse effects associated with hydromorphone use in pediatric patients.

Adverse Effect	Reported Frequency *	Clinical Relevance	Main Clinical Considerations
Sedation	Common	High	Dose-dependent; may be more pronounced than with morphine; requires close monitoring, particularly in postoperative and PICU settings
Respiratory depression	Rare but serious	Very high	Usually associated with cumulative sedative burden or concomitant sedatives rather than hydromorphone alone
Nausea	Common	Moderate	Frequently reported, especially during PCA; highest incidence within the first 24 h
Vomiting	Common	Moderate	Often associated with nausea; may affect recovery and patient comfort
Pruritus	Common	Low–moderate	Occurs despite minimal histamine release; frequency comparable to morphine
Urinary retention	Uncommon	Moderate	Reported mainly with PCA; no consistent difference compared with morphine
Constipation	Common (with prolonged use)	Moderate	Typical opioid-related effect; more relevant during chronic or prolonged therapy
Neuroexcitatory effects (myoclonus, agitation, delirium)	Uncommon	High	Related to the accumulation of hydromorphone-3-glucuronide, especially in renal impairment or prolonged infusion
Hypotension	Rare	Low–moderate	Less frequently associated with histamine release compared with morphine
Tolerance and physical dependence	With prolonged exposure	High	Observed mainly during long-term or continuous infusions, particularly in PICU settings
Opioid use disorder	Rare (pediatric data limited)	High	Risk related to prolonged exposure and high cumulative doses; pediatric evidence is scarce and largely extrapolated from adult data

Legend: * Frequency categories are qualitative and reflect the heterogeneity and limited size of available pediatric studies. Abbreviations: PCA, patient-controlled analgesia; PICU, pediatric intensive care unit.

**Table 5:** Key future perspectives for hydromorphone use in pediatric patients.

Domain	Current Limitations	Future Directions	Expected Clinical Impact
Evidence quality	Predominance of observational and retrospective studies; limited pediatric RCTs	Well-designed, prospective, multicenter pediatric trials	Stronger evidence base to support clinical decision-making
Age-specific dosing	Dosing often extrapolated from adult data; limited data in infants	Development of age- and weight-specific dosing algorithms	Improved safety and efficacy, particularly in vulnerable age groups
PK/PD	Sparse data on PK/PD variability across pediatric ages	Population PK studies and therapeutic drug monitoring approaches	More precise titration and reduced risk of adverse effects
Neonates and infants (<6 months)	Extremely limited clinical and safety data	Dedicated studies in neonatal and infant populations	Safer opioid use in the most vulnerable patients
Comparative effectiveness	Limited head-to-head comparisons with morphine and fentanyl	Comparative trials within multimodal analgesia protocols	Better positioning of hydromorphone among pediatric opioids
Non-invasive administration routes	Limited experience with intranasal and alternative routes	Expansion of intranasal and other minimally invasive strategies	Faster, safer analgesia in emergency and acute care settings
Long-term safety	Poorly characterized neurodevelopmental and cognitive outcomes	Long-term follow-up and neurodevelopmental outcome studies	Improved understanding of long-term risks
Tolerance, dependence, and OUD *	Pediatric-specific data scarce; reliance on adult extrapolation	Systematic evaluation of risk factors and preventive strategies	Safer long-term opioid exposure and reduced misuse risk
Clinical guidelines	Absence of pediatric-specific recommendations	Development of evidence-based pediatric guidelines	Standardization of practice and improved patient safety

Legend: * Evidence on opioid use disorder is mainly derived from adult populations; pediatric-specific data are extremely limited. Abbreviations: OUD, opioid use disorder; PK/PD, pharmacokinetics/pharmacodynamics.

## Data Availability

No new data were created or analyzed in this study. Data sharing is not applicable.

## References

[1] Murphy A., McCoy S., O’Reilly K., Fogarty E., Dietz J., Crispino G., Wakai A., O’Sullivan R. (2016). A Prevalence and Management Study of Acute Pain in Children Attending Emergency Departments by Ambulance. Prehospital Emerg. Care J..

[2] Aqil M. (2012). In Response to Review Article by Mazoit, J.-X. Local Anesthetics and Their Adjuncts. Pediatric Anesthesia, 22: 31–38. doi: 10.1111/j. 1460-9592.2011.03692.x. Pediatr. Anesth..

[3] Marchetti G., Vittori A., Tortora V., Bishop M., Lofino G., Pardi V., De Marco E.A., Manca G., Inserra A., Caruso R. (2016). Prevalence of Pain in the Departments of Surgery and Oncohematology of a Paediatric Hospital That Has Joined the Project “Towards Pain Free Hospital”. Clin. Ter..

[4] Marchetti G., Vittori A., Cascella M., Mascilini I., Piga S., Petrucci E., Castellano A., Caruso R., Francia E., Stocchi F. (2023). Pain Prevalence and Pain Management in Children and Adolescents in an Italian Third Level Pediatric Hospital: A Cross-Sectional Study. Ital. J. Pediatr..

[5] Rodieux F., Ivanyuk A., Besson M., Desmeules J., Samer C.F. (2022). Hydromorphone Prescription for Pain in Children-What Place in Clinical Practice?. Front. Pediatr..

[6] Lynn A.M., Nespeca M.K., Opheim K.E., Slattery J.T. (1993). Respiratory Effects of Intravenous Morphine Infusions in Neonates, Infants, and Children after Cardiac Surgery. Anesth. Analg..

[7] Corli O., Damia G., Galli F., Verrastro C., Broggini M. (2019). Lack of Efficacy: When Opioids Do Not Achieve Analgesia from the Beginning of Treatment in Cancer Patients. Cancer Manag. Res..

[8] MacDonald N., Der L., Allan S., Champion P. (1993). Opioid Hyperexcitability: The Application of Alternate Opioid Therapy. Pain.

[9] Sjøgren P., Jensen N.-H., Jensen T.S. (1994). Disappearance of Morphine-Induced Hyperalgesia after Discontinuing or Substituting Morphine with Other Opioid Agonists. Pain.

[10] Manfredi P.L., Borsook D., Chandler S.W., Payne R. (1997). Intravenous Methadone for Cancer Pain Unrelieved by Morphine and Hydromorphone: Clinical Observations. Pain.

[11] Ashby M.A., Martin P., Jackson K.A. (1999). Opioid Substitution to Reduce Adverse Effects in Cancer Pain Management. Med. J. Aust..

[12] Vittori A., Petrucci E., Cascella M., Innamorato M., Cuomo A., Giarratano A., Petrini F., Marinangeli F. (2021). Pursuing the Recovery of Severe Chronic Musculoskeletal Pain in Italy: Clinical and Organizational Perspectives from a SIAARTI Survey. J. Pain Res..

[13] White W.L. (2011). Erratum to: Why I Hate the Index Finger. Hand.

[14] Babul N., Darke A.C., Hagen N. (1995). Hydromorphone Metabolite Accumulation in Renal Failure. J. Pain Symptom Manag..

[15] Li Y., Yue X., Liang S., Ren F., Guo Q., Zou W. (2024). Effectiveness and Safety of Hydromorphone Compared to Morphine for Postoperative Analgesia: A Systematic Review and Meta-Analysis. Pain Physician.

[16] Spénard S., Metras M.-E., Gélinas C., Shah V., Doré-Bergeron M.-J., Dekoven K., Marquis M.-A., Trottier E.D., Thibault C., Kleiber N. (2024). Morphine versus Hydromorphone in Pediatrics: A Narrative Review of Latest Indications and Optimal Use in Neonates and Children. Minerva Pediatr..

[17] Baethge C., Goldbeck-Wood S., Mertens S. (2019). SANRA-a Scale for the Quality Assessment of Narrative Review Articles. Res. Integr. Peer Rev..

[18] Abi-Aad K.R., Derian A. (2025). Hydromorphone. StatPearls.

[19] Liu L., Xu M., Wang J., Hu Y., Huang Z. (2025). Research Progress of Hydromorphone in Clinical Application. Physiol. Res..

[20] Smith M.T. (2000). Neuroexcitatory Effects of Morphine and Hydromorphone: Evidence Implicating the 3-Glucuronide Metabolites. Clin. Exp. Pharmacol. Physiol..

[21] Wright A.W., Mather L.E., Smith M.T. (2001). Hydromorphone-3-Glucuronide: A More Potent Neuro-Excitant than Its Structural Analogue, Morphine-3-Glucuronide. Life Sci..

[22] Gagnon D.J., Jwo K. (2013). Tremors and Agitation Following Low-Dose Intravenous Hydromorphone Administration in a Patient with Kidney Dysfunction. Ann. Pharmacother..

[23] Spénard S., Gélinas C., DTrottier E., Tremblay-Racine F., Kleiber N. (2021). Morphine or Hydromorphone: Which Should Be Preferred? A Systematic Review. Arch. Dis. Child..

[24] Angst M.S., Drover D.R., Lötsch J., Ramaswamy B., Naidu S., Wada D.R., Stanski D.R. (2001). Pharmacodynamics of Orally Administered Sustained- Release Hydromorphone in Humans. Anesthesiology.

[25] Gupta S., Sathyan G. (2007). Providing Constant Analgesia with OROS^®^ Hydromorphone. J. Pain Symptom Manag..

[26] Sathyan G., Sivakumar K., Thipphawong J. (2008). Pharmacokinetic Profile of a 24-Hour Controlled-Release OROS Formulation of Hydromorphone in the Presence of Alcohol. Curr. Med. Res. Opin..

[27] Drover D.R., Angst M.S., Valle M., Ramaswamy B., Naidu S., Stanski D.R., Verotta D. (2002). Input Characteristics and Bioavailability after Administration of Immediate and a New Extended-Release Formulation of Hydromorphone in Healthy Volunteers. Anesthesiology.

[28] Moore K.T., St-Fleur D., Marricco N.C., Ariyawansa J., Pagé V., Natarajan J., Morelli G., Richarz U. (2010). Steady-State Pharmacokinetics of Extended-Release Hydromorphone (OROS Hydromorphone): A Randomized Study in Healthy Volunteers. J. Opioid Manag..

[29] Moore K.T., St-Fleur D., Marricco N.C., Ariyawansa J., Pagé V., Natarajan J., Morelli G., Richarz U. (2011). A Randomized Study of the Effects of Food on the Pharmacokinetics of Once-Daily Extended-Release Hydromorphone in Healthy Volunteers. J. Clin. Pharmacol..

[30] Fromm M.F., Eckhardt K., Li S., Schänzle G., Hofmann U., Mikus G., Eichelbaum M. (1997). Loss of Analgesic Effect of Morphine Due to Coadministration of Rifampin. Pain.

[31] Wehrfritz A., Schmidt S., Ihmsen H., Schüttler J., Jeleazcov C. (2022). Long-Term Stability of Hydromorphone in Human Plasma Frozen at −20 °C for Three Years Quantified by LC-MS/MS. Int. J. Anal. Chem..

[32] Twycross R.G. (1988). Opioid Analgesics in Cancer Pain: Current Practice and Controversies. Cancer Surv..

[33] Graham F., Clark D. (2005). The Syringe Driver and the Subcutaneous Route in Palliative Care: The Inventor, the History and the Implications. J. Pain Symptom Manag..

[34] Bloor K., Leese B., Maynard A. (1994). The Costs of Managing Severe Cancer Pain and Potential Savings from Transdermal Administration. Eur. J. Cancer.

[35] Inturrisi C.E. (2002). Clinical Pharmacology of Opioids for Pain. Clin. J. Pain.

[36] Storey P., Hill H.H., St Louis R.H., Tarver E.E. (1990). Subcutaneous Infusions for Control of Cancer Symptoms. J. Pain Symptom Manag..

[37] Bruera E., Brenneis C., Michaud M., MacMillan K., Hanson J., MacDonald R.N. (1988). Patient-Controlled Subcutaneous Hydromorphone versus Continuous Subcutaneous Infusion for the Treatment of Cancer Pain. J. Natl. Cancer Inst..

[38] Lin R., Zhu J., Feng S., Lin S., Fu J., Yao Y., Hong L., Xin M., Chen Z., Cai Z. (2019). Patient Controlled Analgesia (PCA) versus Non-PCA Intravenous Hydromorphone Titration for Severe Cancer Pain: A Randomized, Controlled, Multicenter, Phase III Trial, HMORCT09-1. J. Clin. Oncol..

[39] Dale O., Hjortkjaer R., Kharasch E.D. (2002). Nasal Administration of Opioids for Pain Management in Adults. Acta Anaesthesiol. Scand..

[40] Rudy A.C., Coda B.A., Archer S.M., Wermeling D.P. (2004). A Multiple-Dose Phase I Study of Intranasal Hydromorphone Hydrochloride in Healthy Volunteers. Anesth. Analg..

[41] Tsze D.S., Pan S.S., DePeter K.C., Wagh A.M., Gordon S.L., Dayan P.S. (2019). Intranasal Hydromorphone for Treatment of Acute Pain in Children: A Pilot Study. AM. J. Emerg. Med..

[42] Balyan R., Dong M., Pilipenko V., Geisler K., Vinks A.A., Chidambaran V. (2020). Hydromorphone Population Pharmacokinetics in Pediatric Surgical Patients. Paediatr. Anaesth..

[43] Washington University School of Medicine (2024). A Randomized Controlled Trial to Compare Hydromorphone vs. Fentanyl in Children Undergoing Tonsillectomy Surgery.

[44] Pan Y., Wang Y., Lie D., Liu D., Chen X., Wu Z., Chen L., Wang H., Peng L., Liang H. (2021). Effectiveness of Analgesia with Hydromorphone Hydrochloride for Postoperative Pain Following Surgical Repair of Structural Congenital Malformations in Children: A Randomized Controlled Trial. BMC Anesth..

[45] Merkel S.I., Voepel-Lewis T., Shayevitz J.R., Malviya S. (1997). The FLACC: A Behavioral Scale for Scoring Postoperative Pain in Young Children. Pediatr. Nurs..

[46] Egbuta C., Mason K.P. (2021). Current State of Analgesia and Sedation in the Pediatric Intensive Care Unit. J. Clin. Med..

[47] Hughes C.G., McGrane S., Pandharipande P.P. (2012). Sedation in the Intensive Care Setting. Clin. Pharmacol..

[48] Jacobi J., Fraser G.L., Coursin D.B., Riker R.R., Fontaine D., Wittbrodt E.T., Chalfin D.B., Masica M.F., Bjerke H.S., Coplin W.M. (2002). Clinical Practice Guidelines for the Sustained Use of Sedatives and Analgesics in the Critically Ill Adult. Crit. Care Med..

[49] Ciccozzi A., Pizzi B., Vittori A., Piroli A., Marrocco G., Della Vecchia F., Cascella M., Petrucci E., Marinangeli F. (2022). The Perioperative Anesthetic Management of the Pediatric Patient with Special Needs: An Overview of Literature. Child.

[50] Hopkins R.O., Choong K., Zebuhr C.A., Kudchadkar S.R. (2015). Transforming PICU Culture to Facilitate Early Rehabilitation. J. Pediatr. Intensive Care.

[51] Vet N.J., Kleiber N., Ista E., de Hoog M., de Wildt S.N. (2016). Sedation in Critically Ill Children with Respiratory Failure. Front. Pediatr..

[52] Fonsmark L., Rasmussen Y.H., Carl P. (1999). Occurrence of Withdrawal in Critically Ill Sedated Children. Crit. Care Med..

[53] Saliski M., Kudchadkar S.R. (2015). Optimizing Sedation Management to Promote Early Mobilization for Critically Ill Children. J. Pediatr. Intensive Care.

[54] Devabhakthuni S., Armahizer M.J., Dasta J.F., Kane-Gill S.L. (2012). Analgosedation: A Paradigm Shift in Intensive Care Unit Sedation Practice. Ann. Pharmacother..

[55] Devlin J.W., Skrobik Y., Gélinas C., Needham D.M., Slooter A.J.C., Pandharipande P.P., Watson P.L., Weinhouse G.L., Nunnally M.E., Rochwerg B. (2018). Clinical Practice Guidelines for the Prevention and Management of Pain, Agitation/Sedation, Delirium, Immobility, and Sleep Disruption in Adult Patients in the ICU. Crit. Care Med..

[56] Playfor S.D. (2008). Analgesia and Sedation in Critically Ill Children. Arch. Dis. Child Educ. Pract. ED.

[57] Gommers D., Bakker J. (2008). Medications for Analgesia and Sedation in the Intensive Care Unit: An Overview. Crit. Care.

[58] Martyn J.A.J., Mao J., Bittner E.A. (2019). Opioid Tolerance in Critical Illness. N. Engl. J. Med..

[59] Izrailtyan I., Qiu J., Overdyk F.J., Erslon M., Gan T.J. (2018). Risk Factors for Cardiopulmonary and Respiratory Arrest in Medical and Surgical Hospital Patients on Opioid Analgesics and Sedatives. PLoS ONE.

[60] Reiter P.D., Ng J., Dobyns E.L. (2012). Continuous Hydromorphone for Pain and Sedation in Mechanically Ventilated Infants and Children. J. Opioid Manag..

[61] Harkin M., Miller J.L., Lim S.Y., Neely S.B., Walsh C.K., Johnson P.N. (2021). Conversion From Continuous Infusion Fentanyl to Continuous Infusion Hydromorphone in the Pediatric Intensive Care Unit. Ann. Pharmacother..

[62] Curley M.A.Q., Harris S.K., Fraser K.A., Johnson R.A., Arnold J.H. (2006). State Behavioral Scale: A Sedation Assessment Instrument for Infants and Young Children Supported on Mechanical Ventilation. Pediatr. Crit. Care Med..

[63] Spady D.W., Saunders D.L., Schopflocher D.P., Svenson L.W. (2004). Patterns of Injury in Children: A Population-Based Approach. Pediatrics.

[64] Kircher J., Drendel A.L., Newton A.S., Dulai S., Vandermeer B., Ali S. (2014). Pediatric Musculoskeletal Pain in the Emergency Department: A Medical Record Review of Practice Variation. Can. J. Emerg. Med..

[65] LeMay S., Johnston C., Choinière M., Fortin C., Hubert I., Fréchette G., Kudirka D., Murray L. (2010). Pain Management Interventions with Parents in the Emergency Department: A Randomized Trial. J. Adv. Nurs..

[66] Trottier E.D., Ali S., Doré-Bergeron M.-J., Chauvin-Kimoff L. (2022). Best Practices in Pain Assessment and Management for Children. Paediatr. Child Health.

[67] Le May S., Ali S., Plint A.C., Mâsse B., Neto G., Auclair M.-C., Drendel A.L., Ballard A., Khadra C., Villeneuve E. (2017). Oral Analgesics Utilization for Children With Musculoskeletal Injury (OUCH Trial): An RCT. Pediatrics.

[68] Ali S., Manaloor R., Johnson D.W., Rosychuk R.J., LeMay S., Carleton B., McGrath P.J., Drendel A.L., Pediatric Emergency Research Canada (2021). An Observational Cohort Study Comparing Ibuprofen and Oxycodone in Children with Fractures. PLoS ONE.

[69] Health Canada Recommends That Children and Youth Not Use Cough and Cold Products That Contain Opioids—Canada.Ca. https://recalls-rappels.canada.ca/en/alert-recall/health-canada-recommends-children-and-youth-not-use-cough-and-cold-products-contain.

[70] Givens M., Rutherford C., Joshi G., Delaney K. (2007). Impact of an Emergency Department Pain Management Protocol on the Pattern of Visits by Patients with Sickle Cell Disease. J. Emerg. Med..

[71] Bitter C., Suter-Zimmermann K., Surber C. (2011). Nasal Drug Delivery in Humans. Curr. Probl. Dermatol..

[72] Coda B.A., Rudy A.C., Archer S.M., Wermeling D.P. (2003). Pharmacokinetics and Bioavailability of Single-Dose Intranasal Hydromorphone Hydrochloride in Healthy Volunteers. Anesth. Analg..

[73] From the American Association of Neurological Surgeons (AANS), American Society of Neuroradiology (ASNR), American Society of Neuroradiology (ASNR), Canadian Interventional Radiology Association (CIRA), Congress of Neurological Surgeons (CNS), European Society of Minimally Invasive Neurological Therapy (ESMINT), European Society of Neuroradiology (ESNR), European Stroke Organization (ESO), Society for Cardiovascular Angiography Interventions (SCAI), Society of Interventional Radiology (SIR) (2018). Multisociety Consensus Quality Improvement Revised Consensus Statement for Endovascular Therapy of Acute Ischemic Stroke. Int. J. Stroke.

[74] Saudan S., Habre W., Ceroni D., Meyer P.-A., Greenberg R.S., Kaelin A., von Ungern-Sternberg B.S. (2008). Safety and Efficacy of Patient Controlled Epidural Analgesia Following Pediatric Spinal Surgery. Paediatr. Anaesth..

[75] Birmingham P.K., Suresh S., Ambrosy A., Porfyris S. (2009). Parent-Assisted or Nurse-Assisted Epidural Analgesia: Is This Feasible in Pediatric Patients?. Paediatr. Anaesth..

[76] Salomäki T.E., Leppäluoto J., Laitinen J.O., Vuolteenaho O., Nuutinen L.S. (1993). Epidural versus Intravenous Fentanyl for Reducing Hormonal, Metabolic, and Physiologic Responses after Thoracotomy. Anesthesiology.

[77] Salomäki T.E., Laitinen J.O., Nuutinen L.S. (1991). A Randomized Double-Blind Comparison of Epidural versus Intravenous Fentanyl Infusion for Analgesia after Thoracotomy. Anesthesiology.

[78] Goodarzi M. (1999). Comparison of Epidural Morphine, Hydromorphone and Fentanyl for Postoperative Pain Control in Children Undergoing Orthopaedic Surgery. Paediatr. Anaesth..

[79] Cramer J. (2019). Comparison of Morphine- and Hydromorphone-Containing Patient-Controlled Epidural Analgesia Solutions in Pediatric Postoperative Patients. J. Pediatr. Pharmacol. Ther..

[80] Heard G.G., Lamberti J.J., Park S.M., Waldman J.D., Waldman J. (1985). Early Extubation after Surgical Repair of Congenital Heart Disease. Crit. Care Med..

[81] Lin H., Nie L. (2022). Application of Hydromorphone and Ropivacaine in Ultrasound-Guided Brachial Plexus Block of Children. J. Perianesth. Nurs..

[82] Gajarawala S., Wells A., Watkins E., Rust B., Archambault M. (2020). Intrathecal Hydromorphone as an Analgesia Option for Gynecology Patients. JAAPA.

[83] Vetter T.R., Carvallo D., Johnson J.L., Mazurek M.S., Presson R.G. (2007). A Comparison of Single-Dose Caudal Clonidine, Morphine, or Hydromorphone Combined with Ropivacaine in Pediatric Patients Undergoing Ureteral Reimplantation. Anesth. Analg..

[84] Schaff P.B. (2022). Pediatrics and Narrative Medicine. Pediatr. Res..

[85] Frizzell K.H., Cavanaugh P.K., Herman M.J. (2017). Pediatric Perioperative Pain Management. Orthop. Clin. North Am..

[86] Maxwell L.G., Kaufmann S.C., Bitzer S., Jackson E.V., McGready J., Kost-Byerly S., Kozlowski L., Rothman S.K., Yaster M. (2005). The Effects of a Small-Dose Naloxone Infusion on Opioid-Induced Side Effects and Analgesia in Children and Adolescents Treated with Intravenous Patient-Controlled Analgesia: A Double-Blind, Prospective, Randomized, Controlled Study. Anesth. Analg..

[87] Peters J.W., Bandell Hoekstra I.E., Huijer Abu-Saad H., Bouwmeester J., Meursing A.E., Tibboel D. (1999). Patient Controlled Analgesia in Children and Adolescents: A Randomized Controlled Trial. Paediatr. Anaesth..

[88] Karl H.W., Tyler D.C., Miser A.W. (2012). Controlled Trial of Morphine vs Hydromorphone for Patient-Controlled Analgesia in Children with Postoperative Pain. Pain Med..

[89] Berde C.B., Lehn B.M., Yee J.D., Sethna N.F., Russo D. (1991). Patient-Controlled Analgesia in Children and Adolescents: A Randomized, Prospective Comparison with Intramuscular Administration of Morphine for Postoperative Analgesia. J. Pediatr..

[90] Hong D., Flood P., Diaz G. (2008). The Side Effects of Morphine and Hydromorphone Patient-Controlled Analgesia. Anesth. Analg..

[91] DiGiusto M., Bhalla T., Martin D., Foerschler D., Jones M.J., Tobias J.D. (2014). Patient-Controlled Analgesia in the Pediatric Population: Morphine versus Hydromorphone. J. Pain Res..

[92] Coluzzi F., Caputi F.F., Billeci D., Pastore A.L., Candeletti S., Rocco M., Romualdi P. (2020). Safe Use of Opioids in Chronic Kidney Disease and Hemodialysis Patients: Tips and Tricks for Non-Pain Specialists. Ther. Clin. Risk Manag..

[93] Biancuzzi H., Dal Mas F., Brescia V., Campostrini S., Cascella M., Cuomo A., Cobianchi L., Dorken-Gallastegi A., Gebran A., Kaafarani H.M. (2022). Opioid Misuse: A Review of the Main Issues, Challenges, and Strategies. Int. J. Environ. Res. Public Health.

[94] Glegg S., McCrae K., Kolla G., Touesnard N., Turnbull J., Brothers T.D., Brar R., Sutherland C., Le Foll B., Sereda A. (2022). “COVID Just Kind of Opened a Can of Whoop-Ass”: The Rapid Growth of Safer Supply Prescribing during the Pandemic Documented through an Environmental Scan of Addiction and Harm Reduction Services in Canada. Int. J. Drug Policy.

[95] Kolla G., Pauly B., Cameron F., Hobbs H., Ranger C., McCall J., Majalahti J., Toombs K., LeMaistre J., Selfridge M. (2024). “If It Wasn’t for Them, I Don’t Think I Would Be Here”: Experiences of the First Year of a Safer Supply Program during the Dual Public Health Emergencies of COVID-19 and the Drug Toxicity Crisis. Harm Reduct. J..

[96] Braithwaite V., Fairgrieve C., Nolan S. (2020). Sustained-Release Oral Hydromorphone for the Treatment of Opioid Use Disorder. J. Addict. Med..

[97] Strang J., Groshkova T., Uchtenhagen A., van den Brink W., Haasen C., Schechter M.T., Lintzeris N., Bell J., Pirona A., Oviedo-Joekes E. (2015). Heroin on Trial: Systematic Review and Meta-Analysis of Randomised Trials of Diamorphine-Prescribing as Treatment for Refractory Heroin Addiction. Br. J. Psychiatry.

[98] Felden L., Walter C., Harder S., Treede R.-D., Kayser H., Drover D., Geisslinger G., Lötsch J. (2011). Comparative Clinical Effects of Hydromorphone and Morphine: A Meta-Analysis. Br. J. Anaesth..

[99] O’Connell N. (2019). Clinical Management in an Evidence Vacuum: Pharmacological Management of Children with Persistent Pain. Cochrane Database Syst. Rev..

[100] Eccleston C., Fisher E., Cooper T.E., Grégoire M.-C., Heathcote L.C., Krane E., Lord S.M., Sethna N.F., Anderson A.-K., Anderson B. (2019). Pharmacological Interventions for Chronic Pain in Children: An Overview of Systematic Reviews. Pain.

[101] Vittori A., Di Fabio C., Francia E., Mascilini I., Tarquini R., Cecchetti C., Marchetti G., Marinangeli F., Grimaldi Capitello T., Cascella M. (2026). Advantages of Remimazolam in Pediatric Anesthesia: A Narrative Review. Children.

[102] Simonini A., Brogi E., Cascella M., Vittori A. (2022). Advantages of Ketamine in Pediatric Anesthesia. Open Med..

[103] Pisters T., Akkermans A., de Hingh I.H.J.T., Luyer M.D.P., Scholten H.J. (2026). Methadone as an Additive to Multimodal Analgesia vs. Epidural Analgesia in Open and Minimal Invasive Pancreatic Surgery: A Retrospective Analysis. Anesth. Res..

[104] Meng M., Lv M., Wang L., Yang B., Jiao P., Lei W., Lan H., Shen Q., Luo X., Zhou Q. (2022). Off-Label Use of Drugs in Pediatrics: A Scoping Review. Eur. J. Pediatr..

[105] Cascella M., Cascella A., Monaco F., Shariff M.N. (2023). Envisioning gamification in anesthesia, pain management, and critical care: Basic principles, integration of artificial intelligence, and simulation strategies. J. Anesth. Analg. Crit. Care.

